# Editorial: Organ microenvironment in vascular formation, homeostasis and engineering

**DOI:** 10.3389/fbioe.2022.1130851

**Published:** 2023-01-10

**Authors:** Akiko Mammoto, Tadanori Mammoto, Jonathan W. Song

**Affiliations:** ^1^ Department of Pediatrics, Medical College of Wisconsin, Milwaukee, WI, United States; ^2^ Department of Cell Biology, Neurobiology and Anatomy, Medical College of Wisconsin, Milwaukee, WI, United States; ^3^ Department of Pharmacology and Toxicology, Medical College of Wisconsin, Milwaukee, WI, United States; ^4^ Department of Mechanical and Aerospace Engineering, The Ohio State University, Columbus, OH, United States; ^5^ Comprehensive Cancer Center, The Ohio State University, Columbus, OH, United States

**Keywords:** angiogenesis, microcirculation, vascular engineering, stem cell, mechanical force

The expansion of blood vessel networks by angiogenesis plays key roles in organ morphogenesis and physiological function. Well-organized and functional blood vessels are necessary for transporting nutrients and gas exchange. In addition to these passive roles, endothelial cells (ECs) that line blood vessels secrete angiocrine factors and dictate tissue structures to control organ development, regeneration and repair from injury. Deregulated angiogenesis contributes to various pathologies, including cancer, retinopathy, diabetes, and arthritis (Carmeliet, 2003). Spatiotemporal changes in organ-specific chemical and mechanical microenvironment control vascular formation (Carmeliet, 2005; Mammoto et al., 2013), which is required for organ morphogenesis and homeostasis. Despite recent progress in our understanding of the molecular mechanisms that regulate angiogenesis, the role of organ-specific microenvironment in angiogenesis is not fully understood partly due to complexity of cellular and non-cellular components in the tissues. To further uncover the mechanisms of organ-specific angiogenesis and vascular functions, we need to use interdisciplinary techniques and technologies including organoid system, organ-on-chip system, *ex vivo* approach, omics approach, computational modeling, and advanced imaging system.

This Research Topic “*Organ Microenvironment in Vascular Formation, Homeostasis and Engineering*” has assembled a Research Topic of original research and review articles that integrate biomedical and bioengineering research to address key questions on how organ-specific microenvironments control angiogenesis in physiology and pathology. Enhanced understanding of the mechanisms by which organ-specific microenvironments control vascular formation using these advanced technologies will promote new strategies for organ and tissue regeneration and repair.

## Tissue microenvironment in angiogenesis in health and disease

It is becoming more evident that ECs provide instructive cues that mediate tissue morphogenesis and differentiation. Asrar and Tucker reviewed the origin and migration of craniofacial ECs and how these cells influence the development of craniofacial tissues such as salivary glands, teeth and jaw. In addition to organ development, angiogenesis constitutes an essential part of organ regeneration (Ding et al., 2011; Mammoto and Mammoto, 2019). Mechanical factors such as extracellular matrix (ECM) stiffness, shear stress, and stretching forces control angiogenesis and vascular function (Mammoto and Mammoto, 2019). Among these mechanical factors, Mammoto et al. have focused on the pulmonary artery (PA) pressure that transiently increases during regenerative lung growth after unilateral pneumonectomy (PNX) and demonstrated that increases in PA pressure following PNX control angiogenesis through mechanosensitive transcriptional co-activator, YAP1. Extracellular vesicles (EVs) that contain cargo such as nucleic acids, miRNAs, proteins, and lipids play key roles in autocrine and paracrine signaling and promote tissue regeneration (Kourembanas, 2015). The authors also show that blood vessel formation is stimulated in the fibrin gel containing EVs isolated from post-PNX mouse lung ECs or pressurized ECs, while YAP1 knockdown inhibits the effects, suggesting that increases in PA pressure stimulate angiogenesis through the YAP1 pathway during lung regeneration and EC-derived EVs have potential to stimulate angiogenesis.

Tissue microenvironment also contributes to various pathologies. For example, the crosstalk of tumor cells and non-cancerous cells within the tumor microenvironment is a crucial part of the tumor angiogenesis. Guarino et al. have demonstrated that tumor-derived EVs, which play a key role in autocrine and paracrine signaling involved in tumor angiogenesis (Asare-Werehene et al., 2020), downregulate TRPV4 expression and induce abnormal angiogenesis by activating Rho/Rho kinase/YAP/VEGFR2 pathways. Their results suggest that tumor-derived EVs and TRPV4 are novel targets for vascular normalization and cancer therapy. Holter et al. utilized microscale engineering technology to model and investigate stromal fibroblast cell-EC interactions within the tumor microenvironment. Here they showed that fibroblast secreted CXCL12 can reprogram the tumor microenvironment by potently inducing a leakier endothelium that is hospitable to angiogenesis and tumor cell intravasation.

Strategies for bioengineered blood vessels leverage the factors derived from the tissue microenvironment for creating prevascularized tissue constructs. Shafiee et al.review the cell-based co-culture strategies for tissue engineering prevascularized constructs. Here they emphasize co-culture strategies with endothelial lineage cells with different supporting cells, such as mesenchymal stem cells (MSCs), fibroblasts, and perivascular cells, towards the formation of organized and functional vascular networks. Mykuliak et al. extend this concept by comparing the effects of bone marrow-derived mesenchymal stem/stromal cells (BMSCs) and adipose tissue-derived mesenchymal stem/stromal cells (ASCs) in supporting the formation of mature and interconnected networks in a microfluidic chip. Here they show differences in blood vessel function and morphometrics due to differences in tissue origin of the MSCs. Luo et al. have demonstrated that hyaluronic acid (HA), one of the major ECM components constituting tissue microenvironment, promotes angiogenesis induced by human umbilical-derived MSCs and endothelial colony forming cells (ECFCs) in Matrigel plugs and increases blood perfusion of the ischemic mouse limb; HA supported cell proliferation and migration, and enhanced CD44 expression by downregulating microRNA-139-5p in ECFCs.

One of the primary goals of vascular tissue engineering is to address the mortality and morbidity associated with ischemic diseases. Two papers developed novel experimental models to gain insights into specific aspects of tissue ischemia and reperfusion injury. Willi et al. developed an *ex vivo* intestine tissue-based microfluidic model to investigate acute post-ischemic effects on microvascular stability, remodeling, and collateral flow formation. This model uses intact tissue such that one can readily study the responses of perivascular cells, such as pericytes, due to altered flow conditions and vascular occlusion. Optical tweezers is one of the most widely used approaches for single-molecule and single-cell biophysics. Shao et al. used infrared optical tweezers for a novel application in controlling dynamic reperfusion in subdermal capillaries in mice. Here they demonstrate the capacity of optical tweezers as a non-invasive strategy for manipulating blood flow conditions.

## Advanced *in vitro* systems to mimic tissue microenvironments

Lampejo et al. discuss the role of biomimetic tissue engineered models for advancing our understanding of microvascular physiology. They emphasize the importance of widely adopted biomimetic modeling approaches in incorporating the necessary physiological complexity that reconstitute native environments for investigating microvascular dynamics. They examine the application and future opportunities of biomimetic microvascular models for enabling basic science discoveries and therapeutic evaluation studies by collaborating tissue engineers, physiologists, and vascular biologists.

Anti-angiogenesis agents have been used as anti-cancer drugs due to their combined mode of action in preventing neovascularization and disruption of existing vasculatures in the tumor microenvironment. However, it is challenging to validate the anti-angiogenic properties of these drugs due to lack of proper *in vitro* angiogenesis models comprised of mature and long-lived vascular networks. Yavvari et al. developed a three-dimensional drug-testable *in vitro* angiogenesis system in which human umbilical vein ECs are embedded and sandwiched in the collagen scaffold and co-cultured with human dermal fibroblasts. Using this system, authors have demonstrated that single or combinational anti-angiogenic drugs can be tested to predict the effects of these drugs on the vasculatures *in vivo*.

Recent advances in the organoid systems make them promising models for regenerative medicine, drug testing and developmental biology (Gupta et al., 2021). Induced pluripotent stem cells can be differentiated into kidney organoids that develop nephrons, resembling cellular and architectural complexity in the developing kidney (Takasato et al., 2016). However, these organoid systems have several limitations, such as the limited culture duration, loss of nephrogenic potential, immaturity and lack of vasculature (Nishinakamura, 2019), partly due to the lack of mimicking an *in vivo* microenvironment (Rossi et al., 2018). Since kidneys develop in hypoxia *in vivo*, to make more clinically applicable kidney organoids, Schumacher et al. cultured kidney organoids under physiological hypoxia and found that this condition initiates angiogenesis, leading to enhanced angiocrine factor secretion and improved endothelial patterning. Recapitulating the physiological environment in the organoid systems is important to improve the vascularization of organoids and extend their potential for tissue engineering and drug discovery.

In conclusion, the papers in this Research Topic “*Organ Microenvironment in Vascular Formation, Homeostasis and Engineering*” cover a broad area of research from fundamental understanding of the role of tissue microenvironment in angiogenesis to advancements in the technology that can be leveraged for improved strategies for tissue engineering and development of new treatments for various diseases.

## References

[B1] Asare-Werehene M. Nakka K. Reunov A. Chiu C. T. Lee W. T. Abedini M. R. (2020). The exosome-mediated autocrine and paracrine actions of plasma gelsolin in ovarian cancer chemoresistance. Oncogene 39 (7), 1600–1616. 10.1038/s41388-019-1087-9 31700155 PMC7018662

[B2] Carmeliet P. (2003). Angiogenesis in health and disease. Nat. Med. 9 (6), 653–660. 10.1038/nm0603-653 12778163

[B3] Carmeliet P. (2005). Angiogenesis in life, disease and medicine. Nature 438 (7070), 932–936. 10.1038/nature04478 16355210

[B4] Ding B. S. Nolan D. J. Guo P. Babazadeh A. O. Cao Z. Rosenwaks Z. (2011). Endothelial-derived angiocrine signals induce and sustain regenerative lung alveolarization. Cell 147 (3), 539–553. 10.1016/j.cell.2011.10.003 22036563 PMC3228268

[B5] Gupta N. Dilmen E. Morizane R. (2021). 3D kidney organoids for bench-to-bedside translation. J. Mol. Med. Berl. 99 (4), 477–487. 10.1007/s00109-020-01983-y 33034708 PMC8026465

[B6] Kourembanas S. (2015). Exosomes: Vehicles of intercellular signaling, biomarkers, and vectors of cell therapy. Annu. Rev. Physiol. 77, 13–27. 10.1146/annurev-physiol-021014-071641 25293529

[B7] Mammoto A. Mammoto T. (2019). Vascular niche in lung alveolar development, Homeostasis, and regeneration. Front. Bioeng. Biotechnol. 7, 318. 10.3389/fbioe.2019.00318 31781555 PMC6861452

[B8] Mammoto T. Mammoto A. Ingber D. E. (2013). Mechanobiology and developmental control. Annu. Rev. Cell Dev. Biol. 29, 27–61. 10.1146/annurev-cellbio-101512-122340 24099083

[B9] Nishinakamura R. (2019). Human kidney organoids: Progress and remaining challenges. Nat. Rev. Nephrol. 15 (10), 613–624. 10.1038/s41581-019-0176-x 31383997

[B10] Rossi G. Manfrin A. Lutolf M. P. (2018). Progress and potential in organoid research. Nat. Rev. Genet. 19 (11), 671–687. 10.1038/s41576-018-0051-9 30228295

[B11] Takasato M. Er P. X. Chiu H. S. Little M. H. (2016). Generation of kidney organoids from human pluripotent stem cells. Nat. Protoc. 11 (9), 1681–1692. 10.1038/nprot.2016.098 27560173 PMC5113819

